# 594. Enhancing Burn Resuscitation Communication: Implementation of a Flowsheet for Nursing Handoff

**DOI:** 10.1093/jbcr/irag033.388

**Published:** 2026-04-06

**Authors:** Sherrina Richards, M S N Ed, Abigail J Abernathy, Susan L Smith, Howard G Smith

**Affiliations:** Warden Burn Center at Orlando Health Orlando Regional Medical Center, Orlando, FL; Warden Burn Center at Orlando Health Orlando Regional Medical Center, Orlando, FL; Warden Burn Center at Orlando Health Orlando Regional Medical Center, Orlando, FL; Warden Burn Center at Orlando Health Orlando Regional Medical Center, Orlando, FL; Warden Burn Center at Orlando Health Orlando Regional Medical Center, Orlando, FL

## Abstract

**Introduction:**

Clear and effective communication during burn resuscitation is critical for accurate fluid management, patient safety, and positive clinical outcomes. Research has shown that poor handover communication is a leading contributor to over 60% of sentinel patient events. In the Burn Trauma Intensive Care Unit (BTICU), a common practice for documenting key resuscitation data, such as fluid volumes and urine output, involves writing on patient room doors. This method is vulnerable to errors and miscommunication. Nurses voiced concerns about the accuracy of this data and the challenges it poses in effectively communicating patient progress to the burn team during the acute resuscitation phase. The purpose of this project was to develop a standardized Burn Resuscitation Flowsheet (BRFS) to be used during handoff reports and is accessible to the entire burn team, promoting consistency, accuracy, and improved communication.

**Methods:**

The initial version of the BRFS was developed using the American Burn Association’s quality data definitions as a foundation. Design elements and data points were reviewed during the monthly Burn Quality (BQ) Meeting to gather feedback and ensure alignment with clinical needs. During the BTICU quarterly summit, the flowsheet was introduced to staff, and a survey was conducted to assess nurses’ perspectives on the current documentation process and the relevance of the new tool. Following this input, final revisions were made, and the updated version underwent a second review by the BQ team.

**Results:**

The Acute Burn Resuscitation Pre-Flowsheet Survey was completed by 21 RNS (86%) response rate.

Results included the following key statements:

“There is consistently lack of or inaccurate information translated in hand off”.

“Each nurse does it differently”.

“The current process is messy”.

24% disagree that the practice of recording on the door tracks all the necessary information to record burn resuscitation accurately.

**Conclusions:**

In response to the development and implementation of a standardized BRFS aimed to promote clarity, consistency, and improved handoff communication. Survey findings revealed significant dissatisfaction with the existing process, reinforcing the need for a structured tool. The finalized flowsheet, reviewed and refined through collaborative input from nursing staff and the BQ team, represents a meaningful step toward enhancing the quality and safety of burn resuscitation practices.

**Applicability of Research to Practice:**

Future improvement plans include transitioning the BRFS to an electronic format on the hospital’s SharePoint site to enhance accessibility Additionally, the team aims to begin collecting data to evaluate the impact of the flowsheet on burn resuscitation practices, specifically focusing on patient outcomes within the first 24 hours post-injury.

**Funding for the study:**

N/A.

